# Genome Sequences of Six SARS-CoV-2 Strains Isolated in Morocco, Obtained Using Oxford Nanopore MinION Technology

**DOI:** 10.1128/MRA.00767-20

**Published:** 2020-08-06

**Authors:** Meriem Laamarti, M. W. Chemao-Elfihri, Souad Kartti, Rokia Laamarti, Loubna Allam, Mouna Ouadghiri, Imane Smyej, Jalila Rahoui, Houda Benrahma, Idrissa Diawara, Tarek Alouane, Abdelomunim Essabbar, Samir Siah, Mohammed Karra, Naima El Hafidi, Rachid El Jaoudi, Laila Sbabou, Chakib Nejjari, Saaid Amzazi, Rachid Mentag, Lahcen Belyamani, Azeddine Ibrahimi

**Affiliations:** a Medical Biotechnology Laboratory (MedBiotech), Bioinova Research Center, Rabat Medical and Pharmacy School, Mohammed V University in Rabat, Rabat, Morocco; b Medical Biotechnology Center, Moroccan Foundation for Science, Innovation & Research (MAScIR), Rabat, Morocco; c National Reference Laboratory, Mohammed VI University of Health Sciences (UM6SS), Casablanca, Morocco; d Research Center of Plants and Microbial Biotechnologies, Biodiversity and Environment, Microbiology and Molecular Biology Team, Faculty of Sciences, Mohammed V University in Rabat, Rabat, Morocco; e International School of Public Health, Mohammed VI University of Health Sciences (UM6SS), Casablanca, Morocco; f Laboratory of Human Pathologies Biology, Faculty of Sciences, Mohammed V University in Rabat, Rabat, Morocco; g Biotechnology Unit, Regional Center of Agricultural Research of Rabat, National Institute of Agricultural Research, Rabat, Morocco; h Emergency Department, Military Hospital Mohammed V, Rabat Medical and Pharmacy School, Mohammed V University in Rabat, Rabat, Morocco; i Department of Burns, Mohammed V Military Teaching Hospital/Faculty of Medicine and Pharmacy, Mohammed V University in Rabat, Rabat, Morocco; Queens College

## Abstract

Here, we report the draft genome sequences of six severe acute respiratory syndrome coronavirus 2 (SARS-CoV-2) strains. SARS-CoV-2 is responsible for the COVID-19 pandemic, which started at the end of 2019 in Wuhan, China. The isolates were obtained from nasopharyngeal swabs from Moroccan patients with COVID-19. Mutation analysis revealed the presence of the spike D614G mutation in all six genomes, which is widely present in several genomes around the world.

## ANNOUNCEMENT

Severe acute respiratory syndrome coronavirus 2 (SARS-CoV-2) is classified within the subgenus *Sarbecovirus* and genus *Betacoronavirus* and was first identified in Wuhan, China (1), as the causative agent for COVID-19 disease. Since then, the number of COVID-19 cases has risen dramatically (2).

In Morocco, the first SARS-CoV-2 case was confirmed on 2 March 2020. As of 29 June 2020, the number of cases had reached more than 12,248. To understand SARS-CoV-2 genetic diversity and molecular epidemiology in Morocco, we performed complete genome sequencing using the Oxford Nanopore MinION technology.

In this study, we announce the genome sequences of six SARS-CoV-2 strains isolated from patients in Morocco. The samples were obtained by taking nasopharyngeal swabs from six patients with COVID-19. The viral RNA was extracted directly from the swab using the QIAamp viral RNA minikit (Qiagen, Germany), and the Transcriptor first-strand cDNA synthesis kit (Roche) with random hexamers was used to synthetize the viral cDNA.

The ARTIC v3 primers were used with the Q5 high-fidelity DNA polymerase (New England BioLabs [NEB], USA) for virus DNA enrichment. Amplicons of 400 bp were purified using sample purification beads (SPBs) (Illumina, USA) (3) and then quantified with a Qubit 3.0 fluorometer and used for library preparation.

Sequencing was performed on a MinION MK1C instrument with a ligation sequencing kit (catalog number SQK-LSK109) according to a standard protocol (Oxford Nanopore Technologies [ONT], UK), and the six samples were multiplexed in one run. The R9 flow cell was used and run for 2 h.

The sequence reads generated were between 70,565 and 185,364 (Table 1) of raw data per sample, with average lengths of 454 bp, 455 bp, 455 bp, 452 bp, 455 bp, and 454 bp for strains RMPS-01, RMPS-02, RMPS-03, RMPS-04, RMPS-05, and RMPS-06, respectively. Raw reads were mapped to a SARS-CoV-2 reference genome under GenBank accession number NC_045512.2 using BWA-MEM v. 0.7.17 for single-end reads with default settings (4), and SAM/BAM files were manipulated by SAMtools v. 1.9-11 (5). Variant calling was performed using BCFtools v. 1.9 with “mpileup” (5), and variants were further annotated using SnpEff v. 4.3T (6). The consensus sequences were generated by mapping the variants to the reference genomes using BCFtools (5) and then were submitted to the GISAID database and NCBI (accession numbers are listed in Table 1).

**TABLE 1:** Genome features of six strains of SARS-CoV-2

Strain	GenBank accession no.	NCBI SRA accession no.	No. of raw reads	Genome size (bp)	GC content (%)	Coverage (×)	Mapped read %^*a*^
RMPS-01	MT731285	SRX8633309	71,570	29,903	37.96	870.2	99.93
RMPS-02	MT731292	SRX8633310	185,364	29,903	37.96	2,213.9	98.92
RMPS-03	MT731673	SRX8633311	88,452	29,903	37.96	1,022.5	96.51
RMPS-04	MT731327	SRX8633312	113,813	29,903	37.96	1,291	94.9
RMPS-05	MT731468	SRX8633313	70,565	29,903	37.96	834.5	98.2
RMPS-06	MT731764	SRX8633314	128,615	29,903	37.96	1,291	94.9

a Refers to the percentage of reads from the sequenced sample that align directly to a single region on the reference genome.

The phylogenetic analysis was realized using 250 genome sequences retrieved from the GISAID database. The alignment was performed using MAFFT (7) for fast alignment, and maximum-likelihood trees were inferred with IQ-TREE v. 1.5.5 under the generalized time-reversable (GTR) model (8), implemented via the pipeline provided by Augur (github.com/nextstrain/augur). The generated tree was visualized using FigTree 1.4.3 (http://tree.bio.ed.ac.uk/software/figtree). Major clades were defined by amino acid and/or nucleotide substitutions and were matched to the Nextstrain nomenclature (9) (https://nextstrain.org/ncov).

The size of the consensus sequences was similar to that of the Wuhan-Hu-1 reference genome (GenBank accession number NC_045512.2) and was 29,903 bp with a mean coverage ranging from 843.5× to 2,213×. The strain details are found in Table 1.

**FIG 1:**
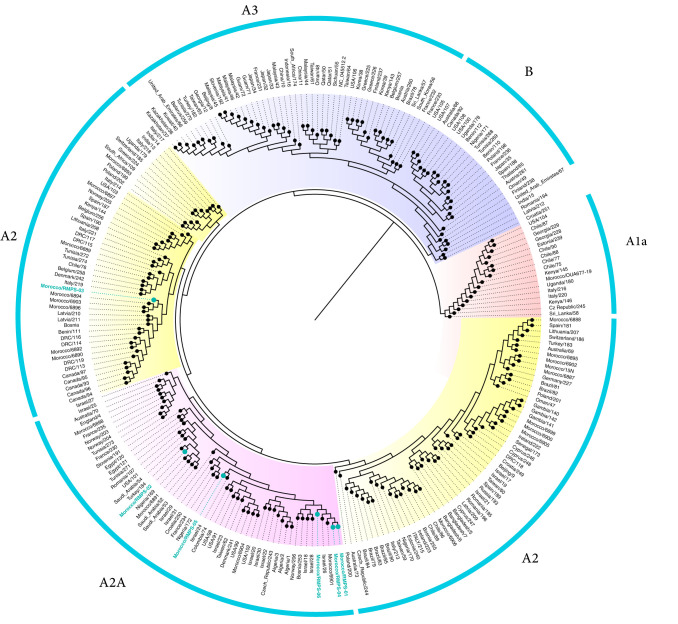
Phylogenetic tree of six SARS-CoV-2 genomes from Morocco. We are currently sequencing more genomes from Morocco to further investigate the spread of COVID-19 and to monitor the evolution of SARS-CoV-2 in Morocco.

We detected 16 different variants in the 6 analyzed genomes. All the genomes shared four mutations, namely, two synonymous (F924F and L4715L), one nonsynonymous (D614G), and one intergenic (241C>T). Only one nonsynonymous mutation was detected (D614G) in the spike protein, which is known as the most prevalent variant worldwide (10), and it is also associated with the emergence of clade A2, which includes all Moroccan strains sequenced in this study (Fig. 1). This mutation was already associated with the observed transmission increase in the United States (10–12).

### Data availability.

The reads of the six SARS-CoV-2 strains were deposited in DDBJ/ENA/GenBank under the SRA accession numbers SRR12109250, SRR12109251, SRR12109252, SRR12109253, SRR12109254, and SRR12109255. The consensus sequences were also deposited in GenBank under the accession numbers MT731285, MT731292, MT731673, MT731327, MT731468, and MT731764.
